# Bœnninghausen’s Therapeutic Pocket-Book

**Published:** 1891-02

**Authors:** 


**Bœnninghausen's Therapeutic Pocket-Book** for homœopathic physicians to use at the bedside and in the study of the Materia Medica. A new American edition. By Dr. Timothy Field Allen. Philadelphia: The Hahnemann Publishing House, 1891. Price, $4.00.

The celebrated *Repertory* of Bœnninghausen has been so many years out of print and copies have become so scarce and dear that the younger generation of homœopathic physicians have scarcely any idea what it is like, and no opportunity to use it. Yet it is the volume above all others, after the *Materia Medica*, upon which the old guard of Hahnemannian homœopathists depended in their daily practice, and which, more than anything else, helped to bring about their successes.

Dr. Allen, the author of the great *Encyclopædia of Materia Medica*, has, therefore, conferred a very obvious benefit upon the profession by once more bringing this great *Repertory* within reach of the whole profession. He has not rested content with simply reproducing the work as it originally appeared, but has added seventeen remedies to the list, making a total of one hundred and forty-two remedies in all. The values of the remedies are made evident by four different styles of type, just as in the original.

The rubrics seem to us somewhat changed in a number of instances, and some change in their order of occurrence is noticeable. Still these changes serve only to increase the convenience of the book.

The work is excellently printed, the pages are small, and binding is in limp leather, making it available for the pocket. Bœnninghausen's original preface is also included, and thus the work is complete.

We recommend it to the whole profession, and have no doubt the edition will be quickly exhausted. W. M. J.

